# Association of clinical knowledge with patient care ownership among resident physicians: a nationwide cross-sectional study in Japan

**DOI:** 10.1186/s12909-025-06694-x

**Published:** 2025-01-16

**Authors:** Hirohisa Fujikawa, Hidetaka Tamune, Yuji Nishizaki, Taro Shimizu, Yu Yamamoto, Kiyoshi Shikino, Miwa Sekine, Hiroyuki Kobayashi, Yasuharu Tokuda

**Affiliations:** 1 Center for General Medicine Education, School of Medicine, Keio University, 35 Shinanomachi, Shinjuku-ku, Tokyo, 160- 8582 Japan; 2 Department of Medical Education Studies, International Research Center for Medical Education, Graduate School of Medicine, The University of Tokyo, Bunkyo-ku, Tokyo, Japan; 3 Department of Psychiatry and Behavioral Science, Juntendo University Graduate School of Medicine, Bunkyo-ku, Tokyo, Japan; 4 Division of Medical Education, Juntendo University School of Medicine, Bunkyo-ku, Tokyo, Japan; 5 Department of Diagnostic and Generalist Medicine, Dokkyo Medical University Hospital, Mibu-machi, Shimotsuga-gun, Tochigi, Japan; 6 Division of General Medicine, Center for Community Medicine, Jichi Medical University, Shimotsuke, Tochigi Japan; 7 Department of Community-Oriented Medical Education, Graduate School of Medicine, Chiba University, Chiba, Chiba Japan; 8 Department of Internal Medicine, Mito Kyodo General Hospital, University of Tsukuba, Mito, Ibaraki Japan; 9 Muribushi Okinawa for Teaching Hospitals, Urasoe, Okinawa Japan; 10 Tokyo Foundation for Policy Research, Minato-ku, Tokyo, Japan

**Keywords:** Clinical knowledge, Medical knowledge, Patient care ownership, Medical resident, Resident physician

## Abstract

**Purpose:**

Both clinical knowledge and patient care ownership (PCO) are crucial to the provision of quality patient care and should be acquired during training. However, the association between these two concepts is under-examined. Here, we conducted a nationwide cross-sectional study to investigate the association between clinical knowledge and PCO among resident physicians in Japan.

**Methods:**

From January 17 to March 31, 2024, we conducted an anonymous, online, self-administered survey for a series of PCO research projects. The survey targeted medical residents who took the General Medicine In-Training Examination (GM-ITE). The mean of the Japanese version of the PCO Scale was used as outcome variable. The primary explanatory variable was total GM-ITE score, while the secondary explanatory variables were GM-ITE category scores in medical knowledge. We conducted multivariable linear regression analysis, controlling for postgraduate years, sex, number of assigned inpatients, weekly working hours, type of hospital, and size of hospital.

**Results:**

We included 1836 participants in our statistical analysis. Multivariable linear regression analysis revealed that after adjustment for possible confounders, GM-ITE total scores showed a significantly negative association with PCO in the highest score quartile (adjusted mean difference − 0.20, 95% confidence interval (CI) -0.33 to -0.07, compared with the lowest score quartile). Additionally, after controlling for possible confounding factors, scores for symptomatology and clinical reasoning showed a dose-dependent negative association with PCO (adjusted mean difference − 0.17, 95% CI -0.30 to -0.03 for the highest score quartile compared with the lowest score quartile). No significant dose-dependent associations were found for the other categories.

**Conclusions:**

These findings suggest the presence of potential challenges in the simultaneous fostering of clinical knowledge and PCO during residency training. This underscores the need for educators to actively engage in the reconsideration of current postgraduate training strategies, with the aim of effectively cultivating both clinical knowledge and PCO among medical residents.

## Background

Medical professionalism is given considerable attention internationally [1, 2]. In 2002, several authors published the Physician Charter, which outlined three fundamental principles and ten professional responsibilities [3, 4]. Since then, the charter has garnered widespread acceptance among physicians and been endorsed by over 100 international organizations, signifying a growing recognition of the importance of medical professionalism [5]. Also in Japan, medical professionalism receives substantial attention and is listed as a competency that should be consistently developed in medical education, both before and after graduation [6–9]. The Model Core Curriculum for Medical Education has served as the undergraduate medical education guideline across Japan [9]. The latest version of the Model Core Curriculum lists the ten basic qualities and abilities required for physicians as follows: Professionalism, Generalism, Lifelong Learning, Research, Problem-Solving, Information Technology, Clinical Skills, Communication, Interprofessional Collaboration, and Medicine in Society [6, 7]. The objectives set for the early residency program include Professionalism, Nine competencies, and Basic clinical tasks [8]. Most of the nine competencies are consistent with the qualities and abilities described in the Model Core Curriculum [9]. Thus, medical professionalism is a crucial competency that should be nurtured in medical education.

Patient care ownership (PCO) is a key element of medical professionalism [10, 11], and is crucial for quality patient care [12, 13]. It is defined as a cognitive-affective state that develops as a result of the individual’s knowledge and management of patients and emotional investment in the relationship with them [11–13]. PCO is associated with better clinical skills, better patient care, and greater patient satisfaction [12, 14]. Accordingly, it is essential to better patient care and should be acquired during training [11, 15]. A recent scoping review identified several factors influencing PCO, including logistical concerns, the physician’s personal attributes or attitudes, and socially or organizationally constructed expectations [14]. There is concern that PCO may be undermined by physician working hour restrictions internationally, including in Japan [12, 16–18]. However, no effective intervention measures to optimize PCO have been established, and the search for a solution continues globally.

PCO was conventionally described as “the philosophy that one knows everything about one’s patient and does everything for them” [19]. However, the comprehension of PCO is currently undergoing a process of expansion and enrichment beyond this initial understanding [20]. Recent studies suggest that PCO consists of a range of dimensions [14, 20, 21]. A qualitative study by McLaren et al. identified seven core elements of PCO: advocacy, autonomy, commitment, communication, follow-through, knowledge, and teamwork [11]. In their qualitative study, Cowley et al. showed that PCO was composed of multiple factors, namely advocacy for the patient, communication and care coordination, decision-making about the therapeutic plan, follow-through in completing tasks related to patient care, acquiring knowledge about the patient, leadership, doing more than the minimum required, seeing oneself as ultimately responsible, sense of responsibility, serving as the primary provider, taking initiative, and providing the best care for the patient [22]. Thus, PCO is a multifaceted concept that is crucial for quality patient care.

Clinical knowledge is also indispensable to the provision of good patient care [23]. The requisite knowledge should be learned during residency, as listed by the Accreditation Council for Graduate Medical Education (ACGME) as one of the six competencies that residents should acquire [24]. Clinical knowledge is also accorded considerable importance in the context of Japanese medical education [7–9]. The ability to correctly identify symptoms and their underlying causes and to provide appropriate therapy is directly linked to the physician’s depth of clinical knowledge [25]. Inadequate clinical knowledge is one of the most common causes of medication error [26]. Gray et al. found that higher diagnostic knowledge among U.S. general internists was associated with a significant reduction in subsequent adverse outcomes whose cause was at risk for diagnostic error [27]. Accordingly, resident physicians should seek to acquire greater clinical knowledge to ensure better patient care.

Therefore, both clinical knowledge and PCO are crucial to the provision of quality patient care and should be acquired during training. However, this requirement gives rise to the following question: “Is the amount of clinical knowledge associated with PCO?” Indeed, the association between these two concepts is unknown, and rationales justifying either a positive or negative result might be considered. One hypothesis is that medical residents with greater clinical knowledge work hard for their patient, indicating greater PCO. A second hypothesis is that resident physicians with a greater amount of clinical knowledge spend much of their time studying at their own desk and have less time to attend patients at the bedside, indicating a lower level of PCO. Whatever the case, we are unaware of any previous study of this association. This lack of knowledge prevents any development of an in-depth understanding of clinical knowledge and PCO.

Thus, examination of the association between clinical knowledge and PCO should be considered a high priority. Here, with the aim of providing deep insight into postgraduate medical education in resident physicians, we examined this research question using the General Medicine In-Training Examination (GM-ITE), a nationwide in-training examination of clinical knowledge in Japan that is taken by over half of all residents [28, 29].

## Methods

### Design, setting, and participants

This nationwide cross-sectional study was conducted as part of a series of investigations of PCO conducted from January 17 to March 31, 2024. An online, anonymous, self-administered questionnaire was distributed to GM-ITE examinees. The examinees were early residency trainees in postgraduate year 1 (PGY-1) and PGY-2. The participants were asked to complete the questionnaire immediately after they had completed the GM-ITE. The GM-ITE is an examination designed to assess clinical knowledge. A comprehensive review of the GM-ITE exam questions was conducted to ensure that the GM-ITE assessment does not include items related to PCO.

Prior to participation in the study, the participants read the research description document and provided informed consent to participate in the study. The document included an explanation of the anonymous and voluntary nature of the study. We included only participants who showed a willingness to participate in our study.

### Measures

#### Outcome variable: PCO

Outcome variable of the study was PCO, as assessed using the Japanese version of the PCO Scale (J-PCOS) [30–32]. The J-PCOS was well validated in previous research [32]. It has 13 items, each of which is rated on a 7-point Likert scale (from 1 = strongly disagree to 7 = strongly agree) [32]. In this study, the J-PCOS was scored as the mean of the 13 items and accordingly ranged from 1 to 7, with a higher score indicating greater PCO.

#### Explanatory variable: GM-ITE scores

In this study, the primary explanatory variable was total GM-ITE score. The GM-ITE is an in-training examination to assess medical knowledge using a methodology similar to that of the U.S. Residency Internal Medicine In-Training Examination [33]. The GM- ITE was developed by the Japan Institute for Advancement of Medical Education Program (JAMEP; nonprofit organization) in 2011 and is now taken by more than half of the resident physicians in Japan [28, 29]. The GM-ITE is 2 h long and consists of 80 multiple-choice questions in several domains. A previous study showed that the GM-ITE score is a validated measure for evaluating medical knowledge [34]. Total score for the examination ranges from 0 to 80, with higher scores indicating better clinical knowledge.

The GM-ITE consists of four categories, namely medical interview and professionalism (score range: 0–8), physical examination and clinical procedure (score range: 0–18), symptomatology and clinical reasoning (score range: 0–18), and detailed disease knowledge (score range: 0–36). In this study, we adopted the scores of these four categories as secondary explanatory variables.

#### Covariates

We chose covariates with reference to previous research [14, 30, 31, 35–37]. We included covariates for PGY, sex, number of assigned inpatients, working hours per week, hospital type, and hospital size.

### Statistical analysis

First, we report descriptive statistics, with continuous data as means and standard deviations and categorical data as frequencies and percentages.

Second, to investigate whether there was a significant association between GM-ITE score and PCO, we performed multivariable linear regression analysis. Prior to analysis, we calculated the intraclass correlation coefficient (ICC) to check for a clustering effect [38]. The ICC for this dataset was less than 10% for the outcome variable (i.e., J-PCOS scores). We therefore concluded that institutional setting was not responsible for the variation in the outcome variable [38], and chose to conduct a conventional analysis using multivariable linear regression. We conducted this analysis with control for PGY, sex, number of assigned inpatients, weekly working hours, type of hospital, and size of hospital. We categorized the total GM-ITE scores and category scores into quartile groups.

Previous literature on sample size indicated the need for a sample size per explanatory variable of ≥ 20 for linear regression analysis [39]. Considering our regression model with 19 explanatory variables, the minimum sample size was estimated to be 380. We used a two-sided significance level of *p* < 0.05. Complete case analysis was chosen. All statistical analysis was performed using SPSS version 29.0 (IBM Corp).

### Ethical considerations

We obtained ethical approval from the Ethical Review Committee of the JAMEP (approval no. 23 − 21).

## Results

Of the 9106 GM-ITE examinees, 2050 consented to participate in our study. Of these, 214 had missing data. Accordingly, we included 1836 participants in our analysis. Table 1 shows the profile of the 1836 participants. 1260 (68.6%) were male, and 1488 (81.0%) worked in community hospitals.

**Table 1:** Participant profile (*N* = 1836)

	Value
Sex, n (%)	
Female	576 (31.4)
Male	1260 (68.6)
PGY, n (%)	
1	901 (49.1)
2	935 (50.9)
Number of assigned inpatients, n (%)	
0–4	674 (36.7)
5–9	961 (52.3)
10–14	153 (8.3)
≥ 15	48 (2.6)
Hospital type, n (%)	
Community hospital	1488 (81.0)
University hospital	234 (12.7)
University branch hospital	114 (6.2)
Hospital size, n (%)	
< 400 beds	511 (27.8)
400–499 beds	389 (21.2)
500–699 beds	561 (30.6)
≥ 700 beds	375 (20.4)
Working hours per week, n (%)	
< 50 h	395 (21.5)
≥ 50 to < 55 h	360 (19.6)
≥ 55 to < 60 h	285 (15.5)
≥ 60 to < 70 h	367 (20.0)
≥ 70 to < 80 h	171 (9.3)
≥ 80 to < 90 h	164 (8.9)
≥ 90 h	94 (5.1)
J-PCOS^a^, mean (SD)	4.84 (0.99)
GM-ITE scores, mean (SD)	
Total^b^	45.21 (7.32)
Medical interview and professionalism^c^	5.78 (1.26)
Physical examination and clinical procedure^d^	8.55 (2.39)
Symptomatology and clinical reasoning^e^	10.17 (2.32)
Detailed disease knowledge^f^	20.71 (3.98)

*Abbreviations* J-PCOS = Japanese version of the Patient Care Ownership Scale; PGY = postgraduate year; SD = standard deviation

^a^ Ranging from 1 to 7, with higher scores indicating higher PCO

^b^ Ranging from 0 to 80, with higher scores indicating greater clinical knowledge in this category

^c^ Ranging from 0 to 8, with higher scores indicating greater clinical knowledge in this category

^d^ Ranging from 0 to 18, with higher scores indicating greater clinical knowledge in this category

^e^ Ranging from 0 to 18, with higher scores indicating greater clinical knowledge in this category

^f^ Ranging from 0 to 36, with higher scores indicating greater clinical knowledge in this category

We then performed multivariable linear regression analysis to investigate whether GM-ITE score was associated with PCO (Table 2). After controlling for possible confounders, a dose-dependent association was not observed. However, GM-ITE total score showed a significant negative association with PCO in the highest score quartile (adjusted mean difference − 0.20, 95% confidence interval (CI) -0.33 to -0.07, compared with the lowest score quartile). Score for the category of symptomatology and clinical reasoning was negatively associated with PCO in a dose-dependent manner, after adjustment for possible confounders (i.e., higher category scores were associated with decreasing levels of PCO) (adjusted mean difference − 0.17, 95% CI -0.30 to -0.03 for the highest score quartile, compared with the lowest score quartile). Although a dose-dependent association was not detected, score for the category of medical interview and professionalism showed a significant negative association with PCO in the second and third score quartiles.

**Table 2:** Association between GM-ITE scores and PCO (*N* = 1836)

Exposure	Unadjusted mean difference (95% CI)	Adjusted^a^ mean difference (95% CI)
**GM-ITE total scores**		
Q1 (score range: 0–40)	Ref.	Ref.
Q2 (score range: 41–45)	-0.07 (-0.19 to 0.06)	-0.10 (-0.22 to 0.03)
Q3 (score range: 46–50)	0.03 (-0.09 to 0.16)	-0.04 (-0.16 to 0.09)
Q4 (score range: 51–80)	-0.09 (-0.22 to 0.04)	-0.20 (-0.33 to -0.07)**
**GM-ITE category scores**		
Medical interview and professionalism		
Q1 (score range: 0–5)	Ref.	Ref.
Q2 (score range: 6)	-0.13 (-0.24 to -0.03)*	-0.12 (-0.23 to -0.02)*
Q3 (score range: 7)	-0.20 (-0.32 to -0.08)**	-0.19 (-0.31 to -0.07)**
Q4 (score range: 8)	-0.04 (-0.21 to 0.14)	-0.01 (-0.19 to 0.16)
Physical examination and clinical procedure		
Q1 (score range: 0–7)	Ref.	Ref.
Q2 (score range: 8)	0.05 (-0.09 to 0.18)	0.03 (-0.10 to 0.16)
Q3 (score range: 9–10)	-0.02 (-0.13 to 0.10)	-0.05 (-0.16 to 0.06)
Q4 (score range: 11–18)	0.00 (-0.13 to 0.13)	-0.09 (-0.21 to 0.04)
Symptomatology and clinical reasoning		
Q1 (score range: 0–9)	Ref.	Ref.
Q2 (score range: 10)	-0.15 (-0.29 to -0.02)*	-0.14 (-0.27 to -0.00)*
Q3 (score range: 11–12)	-0.09 (-0.20 to 0.02)	-0.14 (-0.25 to -0.03)*
Q4 (score range: 13–18)	-0.10 (-0.23 to 0.04)	-0.17 (-0.30 to -0.03)*
Detailed disease knowledge		
Q1 (score range: 0–18)	Ref.	Ref.
Q2 (score range: 19–21)	-0.02 (-0.14 to 0.10)	-0.03 (-0.15 to 0.08)
Q3 (score range: 22–23)	-0.03 (-0.16 to 0.11)	-0.09 (-0.23 to 0.04)
Q4 (score range: 24–36)	0.00 (-0.12 to 0.13)	-0.09 (-0.21 to 0.04)

*Abbreviations* CI = confidence interval; GM-ITE = General Medicine In-Training Examination; PCO = patient care ownership; Q = quartile; Ref. = reference category

** *p* < 0.01 * *p* < 0.05

^a^ Adjusted for postgraduate years, sex, the number of assigned inpatients, weekly working hours, type of hospital, and size of hospital

## Discussion

This nationwide cross-sectional study of resident physicians in Japan showed that clinical knowledge of symptomatology and clinical reasoning was negatively associated with PCO. No clear trend was seen in the associations between total GM-ITE score and PCO or between other GM-ITE category scores and PCO, although a negative relationship with PCO was seen for some quartile groups. As noted in the Background, clinical knowledge and PCO are both critical competencies that have been emphasized in recent medical education [7–15]. Accordingly, the findings of this study offer profound insights to postgraduate medical educators that will encourage them to rethink strategies to nurture both clinical knowledge and PCO among medical trainees.

The finding of a negative association between clinical knowledge of symptomatology and clinical reasoning and PCO provides cautionary evidence for postgraduate medical education. Although precise mechanism is unclear, we suggest two possible mechanisms. First, resident physicians are required to develop a range of competencies, and that the acquisition of other competencies in the rush to learn clinical knowledge may be challenging. For example, the ACGME in the U.S. listed the following six core competencies: patient care, medical knowledge, practice-based learning and improvement, interpersonal and communication skills, professionalism, and systems-based practice [24]. All of these competencies are indispensable and require a substantial amount of time to develop. However, due to the increasing complexity of medical inpatients and the growing volume of patient data [40], residents in the present era need to complete more indirect patient care (e.g., documentation and managing orders) and spend less time on direct patient care than those in previous eras [41], which could lead to decreased PCO. Second, a negative link between knowledge and PCO could be due to a mechanical conception of caring, something like “fixing a broken mechanism” [42, 43]. The concept of physician care involves not only technical aspects, but also a range of other aspects such as relational aspects and agency [44]. As physicians gain more medical knowledge, particularly highly technical or specialized knowledge, the cognitive load may increase. This could potentially lead to a shift in focus from holistic, patient-centered care toward more technical or procedural concerns and decreased PCO. The fact that the symptomatology and clinical reasoning category appears to require more desk study (vs. practical training) and more cognitive load for residents than other categories may indicate why only this category, and no others, showed a negative association with PCO. However, these are just hypotheses, and further research is required to better understand the mechanism.

Score for the category of medical interview and professionalism demonstrated a significant negative association with PCO in the second and third score quartiles. Nevertheless, no dose-dependent association was identified in this category, and thus, no clear trend in the relationship between the category and PCO could be established. Further research is helpful to ascertain whether a dose-dependent association can truly be excluded.

The findings of this study suggest the possibility that it is difficult to train for clinical knowledge and PCO at the same time. A previous study indicated that evidence-based electronic resources (e.g., UpToDate (UpToDate Inc.)) can serve as support tools for busy resident physicians to obtain answers to clinical questions raised during rounds and to enhance clinical knowledge [45]. Accordingly, it might be better for clinical training hospitals to facilitate access to evidence-based electronic resources so that their affiliated residents can gain clinical knowledge efficiently. Conversely, a scoping review by Kiger et al. revealed that education and program structures that provide trainees with graded patient involvement and responsibility are considered strong drivers for the development of PCO [14]. A recent U.S. study showed that in the present era of increased supervision of trainees due to concerns about patient safety, PCO was developed through having a voice in a range of contributions to patient care, including involvement in decision-making; having the most knowledge about the patient; and communicating with and for the patient [46]. Thus, a range of educational strategies should be used at the same time to nurture both clinical knowledge and PCO. This may be a challenging task, however, given recent regulatory limits placed on working hours in Japan [47, 48]. Supervisors are required to assess each trainee’s level of clinical knowledge and PCO to provide effective training in accordance with their individual competencies.

We should note some limitations of this study. First, the study was conducted under a cross-sectional design; therefore, any causality or direction of associations could not be determined. Future studies to confirm the significance of this study should adopt a longitudinal study design. Second, there is a concern about possible selection bias. The response rate was relatively low. Resident physicians with a lesser amount of clinical knowledge and/or lower level of PCO were unlikely to provide responses to our questionnaire. If so, this could have led to underestimation of the association between clinical knowledge and PCO. Additionally, GM-ITE examinees may by highly motivated trainees. It is therefore advisable to exercise caution in interpreting the findings of this study. Third, the percentage of resident physicians working in university hospitals was below 20%. This characteristic of the participants may have influenced the responses, considering that about half of the medical residents work in university hospitals in Japan. Fourth, although the results of this study showed statistical significance, the clinical and educational relevance of the score difference is unknown. Future studies to determine the cutoff scores of the J-PCOS should be performed, which would in turn validate the significance of our study. Fifth, the survey only covered Japan. A comparison with similar studies conducted outside Japan would facilitate a more profound understanding of the association between clinical knowledge and PCO.

## Conclusions

We conducted a nationwide cross-sectional survey of resident physicians throughout Japan. The results revealed the presence of a negative association between clinical knowledge of symptomatology and clinical reasoning and PCO. In contrast, no clear trend was identified in the associations between total GM-ITE scores and PCO and between other GM-ITE category scores and PCO, albeit that negative associations with PCO were indicated for some quartile groups. Our findings may offer postgraduate medical educators with insights to encourage them to rethink strategies for promoting the development of both clinical knowledge and PCO among medical trainees.

## Data Availability

The corresponding author can provide the data sets generated and analyzed in this study upon reasonable request.

## Abbreviations

ACGME: Accreditation Council for Graduate Medical Education
GM-ITE: General Medicine In-Training Examination
ICC: Intraclass correlation coefficient
J-PCOS: Japanese version of the Patient Care Ownership Scale
JAMEP: Japan Institute for Advancement of Medical Education Program
PCO: Patient care ownership
PGY: Postgraduate year
